# Further evidence of the heterogeneous nature of impulsivity^☆^

**DOI:** 10.1016/j.paid.2014.11.059

**Published:** 2015-04

**Authors:** Amy J. Caswell, Rod Bond, Theodora Duka, Michael J. Morgan

**Affiliations:** a School of Psychology, University of Sussex, Brighton, UK; b Center for Alcohol and Addiction Studies, Brown University, USA; c Sussex Addiction Research and Intervention Centre (SARIC), School of Psychology, University of Sussex, Brighton, UK; d Norwegian Center for Addiction Research, University of Oslo, Norway

**Keywords:** RI, reflection-impulsivity, MI, motor-impulsivity, TI, temporal-impulsivity, ISTfw, Information Sampling Task (fixed win condition), ISTrc, Information Sampling Task (reward conflict condition), MFF20, Matching Familiar Figures Task, DDT, Delay Discounting Task, MCQ, Monetary Choice Questionnaire, SKIP, Single Key Impulsivity Paradigm, SST, Stop Signal Task, BIS-11, Barratt Impulsiveness Scale, GNG, Go/NoGo Task, IMT, Immediate Memory Task, TCIP, Two Choice Impulsivity Paradigm, Impulsivity, Laboratory measures, Factor analysis, Reflection impulsivity, Motor impulsivity, Inhibitory control, Temporal impulsivity, Human

## Abstract

‘Impulsivity’ refers to a range of behaviours including preference for immediate reward (temporal-impulsivity) and the tendency to make premature decisions (reflection-impulsivity) and responses (motor-impulsivity). The current study aimed to examine how different behavioural and self-report measurements of impulsivity can be categorised into distinct subtypes.

Exploratory factor analysis using full information maximum likelihood was conducted on 10 behavioural and 1 self-report measure of impulsivity.

Four factors of impulsivity were indicated, with Factor 1 having a high loading of the Stop Signal Task, which measures motor-impulsivity, factor 2 representing reflection-impulsivity with loadings of the Information Sampling Task and Matching Familiar Figures Task, factor 3 representing the Immediate Memory Task, and finally factor 4 which represents the Delay Discounting Questionnaire and The Monetary Choice Questionnaire, measurements of temporal-impulsivity.

These findings indicated that impulsivity is not a unitary construct, and instead represents a series of independent subtypes. There was evidence of a distinct reflection-impulsivity factor, providing the first factor analysis support for this subtype. There was also support for additional factors of motor- and temporal-impulsivity. The present findings indicated that a number of currently accepted tasks cannot be considered as indexing motor- and temporal-impulsivity suggesting that additional characterisations of impulsivity may be required.

## Highlights

• Impulsivity is not a unitary construct and instead comprises dissociable subtypes.
• Reflection-impulsivity is a distinct and well-defined facet of impulsivity.
• Additional characterisations of motor-impulsivity are required.
• Several tasks purported to index impulsivity should be treated with caution.
• Researchers should employ multiple measures of types of impulsivity simultaneously.

## 1 Introduction

Impulsivity encompasses a range of behaviours that include making premature decisions, preferring immediate gratification and having difficulties inhibiting motor responses. Impulsivity functions as a dimension of normal behaviour, and it is thought that it can be adaptive in certain situations (Dalley, Everitt, & Robbins, 2011). However, it is also well established that it is associated with a number of negative outcomes (Aichert et al., 2012; Schweizer, 2002; Vigil-Colet & Morales-Vives, 2005) and is elevated in many clinical populations (e.g. de Wit, 2009; Winstanley, Dalley, Theobald, & Robbins, 2004; Winstanley, Eagle, & Robbins, 2006).

There is growing consensus that impulsivity is heterogeneous and should not be considered a unitary construct and should instead reflect a variety of behaviours and processes (Evenden, 1999). In laboratory-based research, investigators have focused on two subtypes of behavioural impulsivity: ‘motor’-impulsivity (MI), as a failure to inhibit a behavioural response (also termed inhibitory control) and the failure to delay gratification (which we will term ‘temporal’-impulsivity [TI], also referred to as delay discounting). A third subtype of ‘reflection’-impulsivity (RI), i.e. the tendency to make decisions without gathering or evaluating necessary information, has also been suggested although it received comparatively little attention. Multiple tasks have been designed to index each subtype including the Stop Signal Task (SST), Go/NoGo (GNG) and Immediate Memory Task (IMT) for MI, the Matching Familiar Figures (MFF20) and Information Sampling Task (IST) for RI, and pen-and-paper measures such as the Monetary Choice Questionnaire (MCQ) and experiential tasks including the Single Key Impulsivity (SKIP) and Two Choice Impulsivity Paradigm (TCIP) for TI. Impulsivity can also be indexed using self-report measures (e.g. Kirby & Finch, 2010; Whiteside & Lynam, 2001), including the Barratt Impulsiveness Scale (BIS-11, Patton, Stanford, & Barratt, 1995).

However, despite agreement that impulsivity comprises of multiple subtypes, they are rarely investigated concurrently and multiple tasks are seldom simultaneously administered to the same participants. Researchers typically select a single measure and refer to it as ‘impulsivity’, disregarding the wide array of processes and subtypes contributing to impulsive behaviour. This practice has led to poor characterisation of the structure of impulsivity, and the relationship of the subtypes to one another.

Of the small number of studies attempting to address this, investigators typically find correlations between dependent variables of a task and have also found relationships between tasks indexing the same subtype (e.g. Broos et al., 2012; Dougherty et al., 2009; Reynolds, Penfold, & Patak, 2008), suggesting that the subtypes may be well-defined. In contrast, relationships between subtypes are not uniformly found (e.g. Broos et al., 2012; de Wit, 2009; Messer, 1976; Reynolds et al., 2008) and investigators employing factor analysis procedures have found that measures of TI and MI load onto different factors of impulsivity, providing evidence that these two subtypes may be distinct (Broos et al., 2012; Lane, Cherek, Rhoades, Pietras, & Tcheremissine, 2003; Reynolds, Ortengren, Richards, & de Wit, 2006).

Collectively these studies provide preliminary evidence that the subtypes of impulsivity may be well-defined and differentiated. However these studies are limited by including too few tasks (e.g. Broos et al., 2012; Lane et al., 2003; Reynolds et al., 2006) despite evidence that more detailed classifications of impulsivity are required.

The SST, GNG and IMT are used interchangeably as measures of MI in spite of evidence that the tasks index distinct processes: ‘*action cancellation*’, i.e. the inhibition of a response *during* its execution, on the SST (Dalley et al., 2011; Winstanley, 2011) and ‘*action restraint*’, i.e. the inhibition of a response *before* it has started, on the GNG and perhaps the IMT (Dalley et al., 2011, 2009; Eagle, Bari, & Robbins, 2008; Reynolds et al., 2008; Winstanley, 2011; Winstanley, Olausson, Taylor, & Jentsch, 2010). There is evidence that different neurotransmitters may contribute to the two processes (Eagle et al., 2008; Winstanley et al., 2010) and factor analysis has indicated two distinct factors of MI (Dougherty et al., 2009; Reynolds et al., 2008).

With regards to TI, participants respond differently to experiential versus pen-and-paper measures (Winstanley, 2011), hypothetical versus real rewards (Hinvest & Anderson, 2009; Madden, Bickel, & Jacobs, 1999), monetary versus point rewards (Frederick, Loewenstein, & O’Donoghue, 2002) and also to short versus longer delays (Odum, 2011). However, these paradigms are used interchangeably despite there being no evidence to validate the assumption that they all index the same underlying process.

Research suggests that self-report measures of impulsivity are not analogous with behavioural tasks (Dick et al., 2010). The BIS-11 has been found not to correlate with measures of MI or TI (Lane et al., 2003; Lansbergen, Schutter, & Kenemans, 2007; Reynolds et al., 2006) and investigators predominantly find distinct factors of self-report and behavioural impulsivity (Broos et al., 2012; Havik et al., 2012; Lane et al., 2003; Malle & Neubauer, 1991; Meda et al., 2009). However, there is some evidence that self-report impulsiveness is related to GNG performance (Aichert et al., 2012; Reynolds et al., 2006).

Importantly, no factor analysis studies have included measures of RI despite evidence that it is clinically significant and distinct from other subtypes (e.g. Caswell, Morgan, & Duka, 2013a, 2013b; Morgan, Impallomeni, Pirona, & Rogers, 2006; Morgan, McFie, Fleetwood, & Robinson, 2002). The IST was designed to minimise some of the potential shortcomings of the MFF20 that include confounding by other cognitive processes (Clark, Robbins, Ersche, & Sahakian, 2006; Messer, 1976; Zelniker & Jeffrey, 1976) but although both tasks have been proposed to be analogous measures of RI, there are no factor analysis studies to validate this.

As such, current literature discusses three subtypes of behavioural impulsivity, RI, MI and TI. There is evidence that MI and TI are distinct, although available literature is hampered by limited selection of tasks. This limited selection of tasks is a cause for concern as there is evidence of task differences within proposed subtypes that may have implications for their factor loadings and call into question their validity as indexes of the subtypes. Previous studies have also failed to incorporate RI into factor analysis models of impulsivity, despite evidence of its importance (e.g. Caswell et al., 2013a, 2013b; Morgan et al., 2006; Morgan et al., 2002).

The current study aims to address these issues by investigating the structure of impulsivity using exploratory factor analysis, including measures of RI, to confirm whether impulsivity can be categorised into distinct subtypes. We will include a greater number of putative measures of different subtypes of impulsivity than has been attempted previously, encompassing the three proposed behavioural subtypes of MI, TI and RI (the previously unexplored subtype). The tasks will also include the BIS-11 as a self-report index of impulsivity although it is expected that separate facets of self-report and behavioural impulsivity will be identified.

## 2 Method

### 2.1 Participants

160 (80 m, 80f) student participants at the University of Sussex were recruited, providing informed consent. They were required to be 18–45 years of age, not suffering from any mental illness, not be a heavy smoker (<20 per day), not taking any medication (excluding the contraceptive pill).

Participants were instructed to abstain from the use of illicit recreational drugs for at least 1 week prior to the experiment and from the use of alcohol for at least 12 h prior to the experiments.

### 2.2 Procedure

Participants completed the BIS-11 and the National Adult Reading Task, Alcohol Use Questionnaire and Drug Use Questionnaire followed by a battery of behavioural impulsivity tasks. Tasks were computerised and completed in a random order.

### 2.3 Materials

#### 2.3.1 Self-report and demographic measures

*National Adult Reading Task (NART;* Nelson & O’Connell, 1978): The NART gives an estimate measure of verbal IQ. Participants did not complete the NART if they were dyslexic or second language English (*n *= 23).

*Alcohol Use Questionnaire (AUQ;* Townshend & Duka, 2002): Participants estimate the number of alcohol units they consume per week.

*Drug Use Questionnaire (see* Townshend & Duka, 2005): Participants give details of use for main drug categories. Participants were given a score where 0 = no use; 1 = use of cannabis/hash/marijuana; 3 = use of ecstasy/other drugs.

#### 2.3.2 Impulsivity tasks

##### 2.3.2.1 Self-report impulsivity

*Barratt Impulsiveness Scale, Version 11 (BIS-11;* Patton et al., 1995): The BIS-11 is a 30-item checklist measuring impulsivity. The questionnaire gives a total impulsivity score as well as three subscales of motor-, attentional- and nonplanning-impulsivity.

##### 2.3.2.2 Behavioural impulsivity

*Information Sampling Task (IST;* Clark et al., 2006): Participants open a matrix of boxes to reveal two colours underneath before selecting the colour in the majority. There are two conditions available, each consisting of 10 trials, treated as separate tasks:

(i) *Fixed win (FW)*: Participants win/lose 100 points regardless of number of boxes opened.
(ii) *Reward conflict (RC)*: For every box opened, participants lose 10 points from a bank of 250.

The task gives the probability of being correct that the participant tolerates at the point of decision-making [P(correct)].

*Matching Familiar Figures Task (MFF20;* Cairns & Cammock, 1978; Kagan, Rosman, Day, Albert, & Phillips, 1964): Participants select the one of six visually presented stimuli which is identical to an original image. Participants complete 20 trials.

The task gives a composite Impulsivity score (I-score).

*Stop Signal Task (SST;* Logan, 1994): Participants respond to the direction of visually presented green arrows withholding this response whenever the arrow turns red (the Stop Signal, occurs 25% of trials). Participants complete 120 trials.

The task gives a measure of Stop Signal Reaction Time (SSRTi).

*Go/NoGo (GNG; adapted from* Kim, Iwaki, Imashioya, Uno, & Fujita, 2007): Participants respond whenever a visually presented triangle is pointing upwards (Go trials, occur 60% of trials) withholding this response if a triangle is pointing in another direction (Stop trials, occur 40% of trials). Participants complete 120 trials.

The task gives a measure of the percentage of commission errors to Stop signals.

*Immediate Memory Task (IMT;* Dougherty, Marsh, & Mathias, 2002): Participants press the mouse-button if a 5-digit number string is identical to the preceding string. Participants complete two blocks of 180 s, with a 20 s rest period between blocks.

The task gives a measure of commission errors occurring when a participant makes a premature Go response to a Catch trial (occur 33% of trials).

*Single Key Impulsivity Paradigm (SKIP;* Dougherty, Mathias, Marsh, & Jagar, 2005): Participants press the mouse-button to obtain a point reward. The magnitude of the reward is dependent on the delay between consecutive responses. Participants complete a four-minute trial.

The task gives a measure of average inter-response time.

*Two Choice Impulsivity Paradigm (TCIP;* Dougherty et al., 2005): Participants choose between two shapes representing a smaller-sooner (3 points after 3 s) and larger-later (9 points after 9 s) point rewards. Participants complete 30 trials.

The task gives a measure of the number of smaller-sooner choices.

*Monetary Choice Questionnaire (MCQ;* Kirby, Petry, & Bickel, 1999): A pen-and-paper task on which participants choose between hypothetical large delayed rewards, and smaller more immediate rewards. Participants complete 27 items.

The task gives a measure of discounting of delayed rewards (*k*).

*Delay Discounting Questionnaire (DDT):* The DDT is a variation on the MCQ. The pen-and-paper procedure is identical to the MCQ; participants complete 212 items. Stimuli are presented in a fixed random order.

The task gives a measure of discounting of delayed rewards (*k*).

### 2.4 Statistical analysis

Exploratory factor analysis was conducted using Mplus 7,2 (Muthén & Muthén, 1992–2012). Participant demographics and correlations were analysed using Statistical Package for Social Sciences (SPSS) version 22.

#### 2.4.1 Participant demographics characteristics

Participant demographic information including age, average IQ and alcohol and drug consumption are reported. Gender differences on the tasks were calculated to ensure that there were no differences that may affect the factor structure. Pearson’s correlation coefficient was calculated to identify the relationship between age and impulsivity.

#### 2.4.2 Variable selection

One primary dependent variable was selected per task for the factor analysis. This selection was made in part due to the comparatively small sample size; had the sample been larger, multiple measures from each task could have been included. The selection of one variable per task was also made to maintain consistency between tasks as each task contains a varying number of outcome variables and it is known that factor analysis depend heavily on the number of indicators included per expected factor– including multiple from a select number of measures would have caused imbalances in the factor structure by increasing shared variance (Russo, Leone, Lauriola, & Lucidi, 2008).

Variables included in the factor analysis model were:

BIS-11 total score.
ISTfw P(correct).
ISTrc P(correct).
MFF20 I-score.
DDT mean *k* value.
MCQ mean *k* value.
SKIP average IRT.
TCIP number of impulsive choices.
IMT percentage commission errors.
GNG percentage commission errors.
SST SSRTi.

Data were checked to ensure that any participant who did not understand the task, or displayed inconsistent responding, was excluded from that measure – see Section 3.1 for details of included participants. The SST, SKIP, DDT and MCQ were log 10 transformed to correct issues of non-normality. All variables were coded so that large values indicate increased impulsivity.

#### 2.4.3 Correlations between task

Pearson’s correlation coefficient was calculated to identify the correlations between impulsivity measures selected for the factor analysis. In an additional exploratory analysis, as a variables of interest not included in the primary analysis, correlations between the BIS-11 subscales and the behavioural measures of impulsivity were calculated to identify the relationship between self-report and behavioural measures of impulsivity.

#### 2.4.4 Factor analysis

Exploratory factor analysis (EFA) was conducted to identify the factor structure of impulsivity. The sample size of 160 participants for the 11 items exceeds the suggested minimum ration of 5 participants per item (Gorsuch, 1983).

EFA was carried out using full information maximum likelihood with Geomin oblique rotation.

χ^2^, comparative fit index (CFI), a root mean square error of approximation (RMSEA), and standardized root mean square residual (SRMR) were used to evaluate the fit between the model and the data. CFIs of ⩾0.90 indicate a good fit to the data (Browne & Cudeck, 1993). A RMSEA value <0.05 indicate a good fit to the data (Browne & Cudeck, 1993). Well-fitting models obtain SRMR values <0.05 (Cooke et al., 2013).

As measures of appropriateness of factor analysis, a Kaiser–Meyer–Olkin (KMO) value >.5 indicates acceptable sampling adequacy. A significant result for Bartlett’s test of sphericity indicates that the null hypothesis that the correlation matrix is an identity matrix can be rejected.

## 3 Results

### 3.1 Missing data and exclusions

BIS-11 Data were missing for one participant. SST Data were missing for 3 participants; a further 9 were excluded for GoRTs > 1000msecs, or 100% Stop accuracy as it was assumed that participants had not understood task instructions. GNG Data were missing from 2 participants, one participant was excluded for failing to stop to any Stop signals as it was assumed that they had not understood task instructions. IMT Data were missing for 3 participants. SKIP Data for 3 participants were missing. DDT Data were missing for 1 participant, 14 participants were excluded according to the inclusion criteria (see Johnson & Bickel, 2008). MCQ Data for 3 participants were missing. Full information maximum likelihood (FIML) was used to handle missing data for the factor analysis and therefore all 160 participants were included in the factor analysis (Enders, 2010). Correlational analysis was performed on all included data.

### 3.2 Participant characteristics

The age of participants ranged from 18 to 45 (M 20.85; S.D. 3.79). Estimated verbal IQ ranged from 90 to 124 (M 108; S.D. 7.19). Participants drank on average 17 units of alcohol/week (range 0–72, S.D. 14.39). 40% of participants reported no drug use, 31% reported marijuana use, 29% reported other drug use.

There were no gender differences on any impulsivity measure, see Table 1. There were significant associations between age and the Two Choice Impulsivity Paradigm (*r*(160) = −.173, *p* = .029) and the Delay Discounting Task (*r*(145) = .182, *p* = .029). There were no other correlations between age and any other impulsivity measures [BIS-11, *r*(159) = .067, *p* = .40; SST, *r*(148) = .149, *p* = .07; GNG, *r*(157) = −.017, *p* = .83; IMT, *r*(157) = −.046, *p* = .57; ISTfw, *r*(160) = .135, *p* = .09; ISTrc, *p*(160) = .074, *p* = .35; MFF20, *p*(160) = .043, *p* = .59; SKIP, *p*(157) = −.036, *p* = .65; MCQ, *p*(157) = .082, *p* = .31].

### 3.3 Correlations between tasks

The correlation matrix between primary variables of each task is presented in Table 2a.

Correlations between the BIS-11 subscales and each of the behavioural impulsivity measures are presented in Table 2b.

### 3.4 Factor analysis

A four-factor model was indicated and appeared to fit the data well [*χ*^2^(17) = 15.736, *p *= 0.5426; CFI = 1.000, RMSEA = 0.000, 90%CI = 0.000–0.660; SRMR = 0.031].

The KMO value was .511. Bartlett’s test of sphericity was significant, *x*^2^(55) = 156.06.

**Fig. 1:**
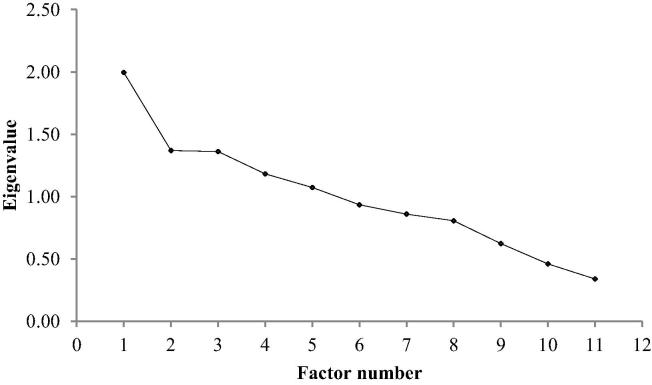
Scree plot indicating number of factors for extraction in the analysis.

The scree plot (Fig. 1) was uninformative and indicated no clear number of factors.

Four factors were retained in the analysis. Factor 1 contained a high loading of the SST. Factor two represents RI with loadings of the IST and MFF20. Factor 3 represents performance on the IMT. Factor 4 represents performance on the DDT and the MCQ. The SKIP, TCIP, GNG and the BIS-11 did not load onto any factors. There were no significant correlations between factors. See Tables 3 and 4 for factor loadings and correlations.

## 4 Discussion

The current study provides important new insights into the structure of impulsivity. The results indicate that impulsivity should not be considered a unitary construct and instead represents a series of independent subtypes. Importantly, the results provide the first factor analysis support for the suggestion of a distinct, well-defined factor of RI. There was also support for the characterisation of behavioural impulsivity into additional factors of MI and TI. The current findings indicated that a number of currently accepted tasks as measurements of MI and TI cannot be considered as indexing these two subtypes and therefore suggest that additional characterisations of impulsivity may be required. Overall, there does not appear to be a strong underlying factor structure; instead, measures purported to index impulsivity typically do not correlate other than in small independent clusters.

The study is the first to implement RI in factor analysis protocols. The results indicated that all putative measures of RI loaded onto a single factor thereby validating these measures and suggesting that, in addition to its clinical significance, RI is distinct from other subtypes of impulsivity. The MFF20 has been criticised as being confounded by other cognitive processes (Block, Block, & Harrington, 1974; Clark et al., 2006; Southgate, Tchanturia, & Treasure, 2008) with the IST developed to circumvent these issues (Clark et al., 2006). However, despite their procedural differences the current study provides the first validation that the two measures index the same primary underlying process.

The results provide evidence that MI can be considered independent from RI and TI; however, it appears that tasks purported to measure MI do not index the same underlying processes. Whilst the SST, IMT and GNG are often used interchangeably, the results indicate that they index different forms of inhibitory control. The SST loaded onto a distinct factor, providing evidence that ‘action cancellation’ (Winstanley, 2011) is dissociable from other forms of inhibitory control. While it has been proposed that the GNG and IMT both index ‘action restraint’ (Winstanley, 2011) the two tasks loaded separately suggesting that the tasks measure different processes. The IMT loaded onto the third factor; on the task, participants must refrain from responding until the correct cue is presented (Winstanley, 2011) and it has been noted that responding on the task is self-generated where participants regulate their behaviour in anticipation of a ‘go’ signal (Winstanley et al., 2010). The results suggest that this form of self-generated responding may be an independent facet of impulsivity, distinct from action cancellation on the SST. The GNG did not load onto any factor suggesting it should be treated with caution as a measure of impulsivity. Overall, the data indicate that types of motor-impulsivity are behaviourally characterisable and are dissociable.

Investigators have developed pen-and-paper and experiential tasks to measure TI, however there has been little research validating the assumption that they index the same process. Our results indicate that pen-and-paper measures (the DDT and MCQ) are analogous and that participants respond consistently despite differences in reward and delay values.

However, neither experiential task loaded onto the factor indicating that they do not index TI as currently understood. Ostensibly, the TCIP and pen-and-paper measures both require participants to select between smaller-sooner and larger-later rewards. However, the tasks differ in the magnitude and type of reward- and delay-values. The comparatively short delays on the TCIP may not have been sensitive to individual differences (Winstanley et al., 2006). The point rewards are received in the laboratory, removing expectations of inflation, future income and the probability of receiving the delayed reward (Frederick, Loewenstein, & O’Donoghue, 2002).

The SKIP is methodologically distinct, utilising a free-operant procedure – the longer participants wait between consecutive responses, the more points they receive. The underlying processes are relatively unexplored; the task correlated with the GNG suggesting that the two may share underlying processes. The results provide evidence that the SKIP and TCIP do not index TI processes as they are currently understood, and that neither are analogous to pen-and-paper measures.

Self-reported impulsivity on the BIS-11 did not load onto any factor. This supports evidence that self-report impulsivity loads separately from behavioural tasks (Broos et al., 2012; Lane et al., 2003; Malle & Neubauer, 1991; Meda et al., 2009) and suggests that the two are heterogeneous. Interestingly, although self-reported impulsivity did not load onto any factor, performance on the IMT was related to BIS-11 total score as well as the nonplanning subscale, the MCQ also correlated with the nonplanning subscale whilst the SST was the only task that correlated with the BIS-11 motor subscale. No behavioural task correlated with the attentional subscale. These correlations suggest that there may be some limited associations between self-reported impulsivity and performance on behavioural tasks.

There were no gender differences in impulsivity indicating that the model applies to both genders, and age did not correlate with any of the measures except for the TCIP and DDT indicating that for the most part impulsivity does not differ with age amongst our sample. All participants were university students however we did not take a measure of income which may have been an important demographic factor of interest. There are further limitations to the analysis which should be discussed. Despite each of the impulsivity tasks providing multiple outcome measures, only the primary impulsivity index was selected from each. Including multiple measures may have provided a more nuanced profile of the constructs under study. For example, the BIS-11 can be categorised into three sub-scores of self-report impulsivity; as these were not included we cannot identify whether any would have loaded onto any of the identified factors, although the lack of consistent correlations between the subscales and the behavioural tasks suggest that they would not. There were two primary reasons for this selection – to address the limited sample size and to avoid imbalances in the number of variables included from each task. The number of subjects is adequate based on our reduced selection of only one variable per task, and selecting multiple variables from each task would have jeopardised this; a larger sample size would have permitted more refined analysis of multiple outcome variables and future studies with greater power are needed to evaluate the sub-scores for each task. The selection of one variable per task was also made to maintain consistency between tasks. Each of the tasks provide a differing number of outcome variables and had we selected multiple from one task (e.g. the BIS-11) we would also have had to select multiple from every other tasks to prevent imbalances in the factor structure (it is known that factor analysis depend heavily on the number of indicators included per expected factor). In light of this we made the decision to select only one per task. Unfortunately, in reality such imbalances are unavoidable, with the Fixed Win and Reward Conflict versions of the IST, and the two pen-and-paper measures of TI being very similar; these methodological overlaps may have implications for the observed factor structure by increasing shared variance.

In summary, the results provide evidence that impulsivity should not be considered a unitary construct, instead consisting of a series of independent subtypes. The data provide compelling support for the suggestion of a distinct, well–defined factor of RI. There was also support for the categorisation of behavioural impulsivity into additional factors of MI and TI. However, the results suggest that a number of currently accepted tasks cannot be considered as indexing these two subtypes, instead indicating that additional characterisations of impulsivity may be important. The results indicate that the IMT represents an additional facet of impulsivity. The data suggest that a number of tasks purported to index impulsivity should be treated with caution, and the results should be used as a basis for investigators in selecting tasks. It is hoped that the results encourage more researchers to implement multiple tasks to index ‘impulsivity’, as opposed to tasks in isolation.

## Figures and Tables

**Table 1:** Gender differences on the impulsivity tasks.

	Males	Females	*p*
BIS-11	65.67 ± 9.92	64.58 ± 10.36	*t*(157) = −.681, *p* = .497
SST	276.50 ± 77.95	267.86 ± 69.56	*t*(146) = −.671, *p* = .503^+^
GNG	3.81 ± 5.37	3.5 ± 4.50	*t*(155) = −.392, *p* = .696
IMT	29.50 ± 11.04	30.62 ± 13.28	*t*(155) = .578, *p* = .564
ISTfw	0.13 ± 0.11	0.11 ± 0.10	*t*(158) = −1.240, *p* = .217
ISTrc	0.23 ± 0.10	0.24 ± 0.09	*t*(158) = .126, *p* = .900
MFF20	−0.26 ± 1.84	0.26 ± 1.63	*t*(158) = 1.921, *p* = .056
SKIP	4.30 ± 5.53	5.36 ± 9.78	*t*(155) = −.579, *p* = .564^+^
TCIP	6.56 ± 6.83	7.58 ± 7.34	*t*(158) = .903, *p* = .368
DDT	0.0311 ± 0.1611	0.0298 ± 0.1529	*t*(143) = −.749, *p* = .455^+^
MCQ	0.0240 ± 0.0351	0.0156 ± 0.0162	*t*(155) = −1.051, *p* = .295^+^

Values are expressed as Mean ± S.D. ^+^Statistics were run on transformed data.

**Table 2a:** Correlation matrix, and significance values, for the 11 tasks.

Correlations	BIS-11	SST	GNG	IMT	ISTfw	ISTrc	MFF20	SKIP	TCIP	DDT
*r(N)*										
SST	.073(147)									
GNG	−.054(156)	.047(148)								
IMT	.177^∗^(156)	−.122(145)	.136(154)							
ISTfw	.054(159)	−.115(148)	.063(157)	.146(157)						
ISTrc	.139(159)	.213^∗∗^(148)	−.012(157)	.124(157)	.401^∗∗^(160)					
MFF20	.000(159)	.088(148)	−.049(157)	−.072(157)	.186^∗^(160)	.119(160)				
SKIP	.086(156)	.010(145)	.236^∗∗^(154)	.041(154)	−.011(157)	.054(157)	−.053(157)			
TCIP	.018(159)	−.068(148)	−.029(157)	−.06(157)	.122(160)	.005(160)	.034(160)	−.056(157)		
DDT	.006(144)	−.042(135)	.037(142)	−.063(142)	.211^∗^(145)	.132(145)	.083(145)	.012(142)	.131(145)	
MCQ	.139(157)	−.078(145)	.103(154)	.011(154)	.184^∗^(157)	.099(157)	−.002(157)	.078(154)	.103(157)	.625^∗∗^(143)

*p*										
SST	.379									
GNG	.499	.573								
IMT	.027^∗^	.145	.092							
ISTfw	.498	.163	.433	.069						
ISTrc	.081	.009^∗∗^	.879	.122	.000^∗∗∗^					
MFF20	1.000	.289	.545	.371	.018^∗^	.135				
SKIP	.287	.910	.003^∗∗^	.611	.888	.504	.508			
TCIP	.821	.412	.723	.456	.125	.954	.673	.489		
DDT	.939	.627	.659	.459	.011^∗^	.115	.320	.890	.115	
MCQ	.083	.351	.202	.893	.021^∗^	.216	.984	.338	.198	.000^∗∗∗^

Values in parentheses refer to sample sizes. ^∗^ indicates significant correlation (^∗^*p* < .05, ^∗∗^*p* < .01, ^∗∗∗^*p* < .001).

**Table 2b:** Correlation matrix, and significance values, between BIS-11 subscales and the behavioural impulsivity measures.

Correlation (N)	SST	GNG	IMT	ISTfw	ISTrc	MFF20	SKIP	TCIP	DDT	MCQ
*r(N)*										
BIS-11 attentional	−018(147)	−.045(156)	.072(156)	−.013(159)	.112(159)	−.069(159)	.050(156)	−.017(159)	−.125(144)	.005(157)
BIS-11 motor	.173^∗^ (147)	−.008(156)	.124(156)	.016(159)	.137(159)	−.021(159)	.046(156)	−.082(159)	.157(144)	.138(157)
BIS-11 Nonplanning	.024(147)	−.069(156)	.206^∗∗^(156)	.106(159)	.085(159)	.069(159)	.099(156)	.114(159)	−.014(144)	.166^∗^(157)

*p*										
BIS-11 attentional	0.829	0.575	0.374	0.874	0.159	0.388	0.538	0.834	0.135	0.95
BIS-11 motor	0.036^∗^	0.917	0.124	0.837	0.084	0.795	0.569	0.304	0.060	0.084
BIS-11 Nonplanning	0.769	0.395	0.01^∗∗^	0.185	0.285	0.387	0.220	0.154	0.867	0.037^∗^

Values in parentheses refer to sample sizes. ^∗^ indicates significant correlation (^∗^*p* < .05, ^∗∗^*p* < .01, ^∗∗∗^*p* < .001).

**Table 3:** Table showing factor loadings after rotation.

	Geomin Rotated Loadings			
	1	2	3	4
BIS-11	0.191	0.025	0.243	0.128
SST	0.686	−0.022	−0.165	−0.012
GNG	0.015	−0.004	0.203	0.083
IMT	−0.012	0.096	0.569⁎	−0.056
ISTfw	−0.055	0.817⁎	−0.005	0.017
ISTrc	0.401	0.517⁎	0.062	0.006
MFF20	0.099	0.275⁎	−0.226	−0.039
SKIP	0.079	−0.056	0.205	0.063
TCIP	−0.107	0.14	−0.143	0.07
DDT	0.025	0.139	−0.133	0.592⁎
MCQ	−0.021	−0.027	0.032	1.049⁎

⁎ Indicates significant loading (⁎*p* < .05).

**Table 4:** Table showing factor correlations.

	Geomin Factor Correlations			
Factor	**1**	**2**	**3**	**4**
**1**	–			
**2**	−0.039	–		
**3**	−0.011	0.172	–	
**4**	−0.028	0.219	0.044	–
